# Impact of flavonoid-rich black tea and beetroot juice on postprandial peripheral vascular resistance and glucose homeostasis in obese, insulin-resistant men: a randomized controlled trial

**DOI:** 10.1186/s12986-016-0094-x

**Published:** 2016-05-13

**Authors:** Dagmar Fuchs, Jean Nyakayiru, Richard Draijer, Theo P. J. Mulder, Maria T. E. Hopman, Thijs M. H. Eijsvogels, Dick H. Thijssen

**Affiliations:** Unilever Research and Development, Vlaardingen, Olivier van Noortlaan 120, PO Box 114, 3130 AC Vlaardingen, The Netherlands; Research Institute for Health Sciences, Department of Physiology, Radboud University Medical Center, Geert Grooteplein-West 32, 6525 GA Nijmegen, The Netherlands; Research Institute for Sport and Exercise Sciences, Liverpool John Moores University, Byrom Street, L3 3AF Liverpool, UK

**Keywords:** Skeletal muscle, Blood flow, Polyphenols, Nitrate, Dietary intervention

## Abstract

**Background:**

Insulin-stimulated muscle blood flow facilitates plasma glucose disposal after a meal, a mechanism that is impaired in obese, insulin-resistant volunteers. Nitrate- or flavonoid-rich products, through their proposed effects on nitric oxide, may improve postprandial blood flow and, subsequently, glucose disposal. To investigate whether a single dose of nitrate-rich beetroot juice or flavonoid-rich black tea lowers postprandial muscle vascular resistance in obese volunteers and alters postprandial glucose or insulin concentrations.

**Method:**

In a randomised, controlled, cross-over study, 16 obese, insulin-resistant males consumed 75 g glucose, which was combined with 100 ml black tea, beetroot juice or control (water). Peripheral vascular resistance (VR), calculated as mean arterial pressure divided by blood flow, was assessed in the arm and leg conduit arteries, resistance arteries and muscle microcirculation across 3 h (every 30-min) after the oral glucose load.

**Results:**

During control, we found no postprandial response in VR in conduit, resistance and microvessels (all *P* > 0.05). Black tea decreased VR compared to control in conduit, resistance and microvessels (all *P* < 0.05). Beetroot juice decreased postprandial VR in resistance vessels, but not in conduit artery and microvessels. Although postprandial glucose response was similar after all interventions, postprandial insulin response was attenuated by ~29 % after tea (*P* < 0.0005), but not beetroot juice.

**Conclusions:**

A single dose of black tea decreased peripheral VR across upper and lower limbs after a glucose load which was accompanied by a lower insulin response. Future studies in insulin-resistant subjects are warranted to confirm the observed effects and to explore whether long-term regular tea consumption affects glucose homeostasis.

**Trial registration:**

The study was registered at clinicaltrials.gov on 30^th^ November 2012 (NCT01746329).

**Electronic supplementary material:**

The online version of this article (doi:10.1186/s12986-016-0094-x) contains supplementary material, which is available to authorized users.

## Background

Impaired glucose homeostasis is strongly related to the development or progression of diabetes mellitus type 2. After a meal, type 2 diabetes mellitus patients are exposed to prolonged elevated concentrations of blood glucose [1]. Skeletal muscle is the principal tissue responsible for insulin-stimulated glucose disposal and therefore significantly contributes to the postprandial regulation of glucose concentrations [2]. The effects of insulin to regulate glucose concentrations are, at least partly, related to reduced microvascular resistance and microvascular recruitment [3]. Indeed, healthy individuals demonstrate an increase in skeletal muscle blood flow after intravenous glucose and oral glucose load or a carbohydrate-rich meal [4]. However, insulin-stimulated vasodilation and glucose uptake are impaired in a step-wise manner in obese individuals and obese type 2 diabetic individuals [4]. Studies suggest that the skeletal blood flow response is blunted to glucose or a carbohydrate-rich meal in obese individuals [4, 5]. These observations highlight the potential clinical relevance of enhancing blood flow and thereby contributing to glucose homeostasis.

Previous studies have provided evidence that endothelial dysfunction is related to impaired postprandial blood flow and glucose responses as well as lower insulin sensitivity [6]. Consumption of tea is associated with lower cardiovascular events [7], possibly through the well-established improvement of vascular endothelial function [8]. A possible underlying mechanism could be the improved bioactivity of the endothelium-derived vasodilator nitric oxide (NO) [9]. Interestingly, a previous study found black tea to protect against the occurrence of the postprandial transient decline in endothelial function and rise in blood pressure (BP) [10]. Based on these direct effects of tea on endothelial function, we hypothesized that tea improves the postprandial blood flow and glucose homeostasis in obese insulin-resistant volunteers.

Another source of NO is beetroot juice which is rich in nitrate that can be transformed in the body to NO [11]. Indeed, recent nutritional studies have demonstrated that food products high in nitrate may improve vascular function. In a recent study, a single dose of oral inorganic nitrate (8 mmol KNO3) lowered BP (-5 mmHg) and arterial stiffness [12]. Moreover, a single dose of beetroot juice attenuated the postprandial impairment of FMD following a standardized mixed meal in healthy overweight and slightly obese men, which may be related to the suggested increase in plasma NO concentrations [13]. Therefore, beetroot juice may also improve postprandial muscle perfusion and, subsequently, glucose homeostasis in obese, insulin-resistant volunteers.

The aim of the present study was to examine the impact of a single dose of flavonoid-rich tea and nitrate-rich beetroot juice, i.e. two potential dietary “sources” of NO, on postprandial skeletal muscle blood flow and glucose homeostasis in obese, insulin-resistant volunteers. For this purpose, we investigated the effect of an oral glucose challenge on leg and forearm blood flow (at conduit, resistance and microvessel level) and glucose metabolism, when consumed with either tea, beetroot juice or control. Based on earlier work, we expected that the blunted postprandial vasodilation in obese, insulin-resistant individuals [4, 5] can be improved by tea and beetroot juice. Moreover, due to the improved postprandial blood flow responses, we expected improved postprandial glucose homeostasis after tea or beetroot juice intake.

## Methods

This study was conducted from December 2012 until April 2013 at the Radboud University Medical Center, Nijmegen, the Netherlands, in accordance with the guidelines laid down in the Declaration of Helsinki (version: 2008) and the Medical Research Involving Human Individuals Act and other guidelines and regulations. All procedures involving human volunteers were approved by the Medical Ethics Committee of Arnhem-Nijmegen, The Netherlands. Written informed consent was obtained from all volunteers prior to participation. Registration no. NCT01746329.

### Volunteers

Sixteen obese, insulin-resistant men were recruited by an advertorial in the local newspaper and on local radio. Volunteers free of diabetes mellitus and/or presence of established cardiovascular disease were eligible. Obesity was defined as a BMI ≥30 kg/m^2^. Insulin resistance was determined by fasting glucose concentrations (>6.1 mmol/l). After submission to the Ethics Committee the protocol was amended by adapting the definition for obesity (BMI ≥30 kg/m^2^ and/or a waist-to-hip ratio of ≥1.00) and for insulin resistance (fasting glucose concentration >5.55 mmol/l) according to recent guidelines [14, 15]. The amendment came into effect after approval by the Ethics Committee. In total, 48 volunteers participated in the screening procedure and 18 volunteers met the inclusion criteria. The main reasons for exclusion were insufficient high fasting glucose concentrations (<5.55 mmol/l) and not being classified as obese. Volunteers treated with drugs that may interfere with the study measurements (e.g. medication affecting BP or glucose metabolism), those who reported alcohol consumption >28 units/week, smokers, and those not willing to comply with the study protocol were excluded.

### Experimental design

The study was conducted using a single-blind, controlled, randomized, cross-over study design. Data collectors, outcome adjudicators, and data analysts were blinded while participants could not be blinded due to the nature of the test products. Volunteers reported trice to the test facility after an overnight fast. After 20-min rest in the supine position, brachial and femoral artery blood flow (echo-Doppler), forearm and leg resistance artery blood flow (venous occlusion plethysmography) and forearm microvascular perfusion (near infra-red spectroscopy) were regularly assessed. After baseline measurements, volunteers randomly received either a single dose of 100 ml tea, beetroot juice or control (hot water), all containing 75 g glucose. Based on the cross-over design of our study with three different interventions, there were six different possibilities regarding the order of interventions. Volunteers were randomly allocated by an independent statistician into six treatment allocations (Williams design, balanced for first order carryover effects). Subsequently, all measurements were repeated (every 30-min) across a three hour period. Blood was taken every 30 min for post-hoc analysis of glucose and insulin. Between the subsequent testing days, a wash-out period of two to seven (on average four) days was provided.

### Intervention

The two active test products were the flavonoid-rich fraction of black tea solids (Lipton Yellow Label tea; Unilever R&D, Vlaardingen, the Netherlands) and a commercially available beetroot juice (Beet-It, James White Drinks Ltd., Ipswich, UK). As the active test products were too different in nature to design a proper placebo product, 100 ml water was used as control to match volume. The tea dose was equal to the same amount of flavonoids found in two cups of black tea (Table 1). The beetroot juice provided 300 mg nitrate. Tea and control were given with 75 g anhydrous glucose (Spruyt Hillen B.V., IJsselstein, the Netherlands) which mimics the blood glucose responses during a meal. As the Beet-It beetroot juice contained 13 g of glucose in the form of free glucose and sucrose, 62 g of anhydrous glucose and 30 ml of water were added to a standard portion of 70 ml beetroot juice.

**Table 1:** Product composition of the flavonoid-rich black tea extract and of beetroot juice

Compounds	Flavonoid-rich black tea extract	Beetroot juice Beet-It
Protein (g)	0.01	2.5
Carbohydrates (g)	0.03	16
Fat (g)	ND	<0.1
Fibre (g)	ND	<0.5
Sodium (mg)	ND	<100
Nitrate (mg)	ND	300
Total Polyphenol (Folin Ciocalteu) (mg)	228.1	68.4
consisting of: (-)-Epicatechin (mg)	2.3	
(-)-Epigallocatechin (mg)	1.1	
(-)-Epigallocatechin gallate (mg)	6.2	
(-)-Epicatechin gallate (mg)	5.0	
Theaflavins (mg)	2.3	
Gallic acid (mg)	3.9	
Flavonol-glycosides (mg)	2.3	
Thearubigins^a^ (mg)	161.9	
Caffeine (mg)	37.1	ND

Product composition is provided per serving, i.e. 442 mg black tea solids and 70 ml beetroot juice, respectively. The composition of Beet-it beetroot juice was provided by James White Drinks Ltd. (Ipswich, UK). Total polyphenol content of tea was determined by Folin Ciocalteu and the content of specific flavonoids by reversed phase HPLC with a Luna Phenyl hexyl column (4.6x250 mm, 5 μm) and a Phenyl propyl guard column (Phenomenex)

^a^Thearubigins were assumed to represent the total polyphenol fraction substracted by catechins, theaflavins, flavonol-glycosides and organic acids (i.e. gallic acid, theogallin and chlorogenic acid)

*ND*, not determined

### Measurements

Volunteers were asked to refrain from spicy meals, nitrate-rich food (i.e. green leafy vegetables or cured meats), tea, alcohol, mouthwash and strenuous exercise for at least 24 h before the measurements. The baseline measurements were performed after a fasting period of at least six hours. During this period volunteers were allowed to drink water *ad libitum*. The measurements were conducted in a temperature-controlled room (22–24 °C) with volunteers in supine position.

#### Glucose homeostasis

Venous blood samples were taken to examine blood glucose and insulin. The homeostasis model assessment (HOMA-IR) was used to examine the degree of insulin resistance, according to: HOMA-IR = [insulin (mU/l) X glucose (mmol/l)]/22.5.

#### Conduit artery blood flow (brachial and femoral artery)

Conduit artery blood flow was determined by echo-Doppler as described previously [16]. In short, a 10-MHz multifrequency linear array probe attached to a high-resolution ultrasound machine (T3000, Terason, Burlington, MA, U.S.A.) was used to image the arteries. Ultrasound parameters were set to optimize longitudinal, B-mode images of the lumen/arterial wall interface. Continuous Doppler velocity assessment was obtained (insonation angle always <60°), which did not vary during the study. For assessment of the brachial artery, volunteers were positioned supine with the right arm extended and immobilized with foam, supported at an angle of approximately 80° from the torso. The brachial artery was scanned 2–5 cm above the antecubital fossa. For the superficial femoral artery, volunteers were positioned supine with the lower leg slightly elevated and supported on a ~15 cm thick foam. The superficial femoral artery was scanned in the proximal third of the thigh, at least 5 cm distal from the bifurcation.

#### Resistance artery blood flow (forearm and leg)

Resistance artery blood flow was assessed using venous occlusion plethysmography (VOP). VOP measures the change in (arm or leg) muscle circumference using a mercury-in-silastic strain gauge during temporarily blockage of venous outflow [17]. The left arm was positioned approximately 5 cm above the level of the heart. A standard BP cuff (10 cm width) was placed around the left upper arm and attached to a rapid cuff inflator (Hokanson, Bellevue, WA, U.S.A.). A mercury-in-silastic strain gauge (Hokanson) was placed at the widest girth of the forearm. The perpendicular distance to the elbow crease was recorded and carefully controlled throughout the experiment. One minute before and during the measurement, the hand circulation was excluded by inflating a cuff (8 cm width) around the wrist to a pressure of 220 mmHg to minimize the contribution of hand skin blood flow. To assess thigh blood flow, a 12 cm width cuff was placed proximally around the left upper leg. The leg was elevated with lower leg resting on a platform 15 cm high. Strain gauge was placed at mid-thigh, ≥10 cm above the patella and > 4 cm below the cuff to avoid displacement of the strain gauge during cuff inflation. For assessment of forearm and thigh resistance artery blood flow, we repeatedly inflated the BP cuffs to 50 mmHg for eight cardiac cycles, followed by deflation for nine cardiac cycles, across a timeframe of five minutes.

#### Microcirculation (forearm)

Near-infrared spectroscopy (NIRS) was used to specifically measure local microvascular muscle blood flow by assessing regional concentration changes in oxyhaemoglobin (O_2_Hb) and desoxyhaemoglobin (HHb), using a continuous wave near-infrared spectrophotometer (OXYMON, Artinis Medical Systems, the Netherlands). Briefly, NIRS is based on the relative transparency of tissue for light in the near infrared region and on the O_2_-dependent absorption changes of haemoglobin and myoglobin. The absorption changes measured directly by the NIRS instrument are converted into estimates of concentration changes of O_2_Hb and HHb by using a modified Lambert-Beer law described in details by Livera et al. [18]. To correct for the light scattering inside the tissue, a differential path length factor of 4.0 was used. The sum of O_2_Hb and HHb reflects changes in blood volume, represented by the total hemoglobin signal (tHb). NIRS measurements in the forearm were obtained by positioning the optodes on the extensor carpi radialis brevis muscles of the forearm at a penetration depth of 15–20 mm. The local muscle blood flow was determined by inflating a blood pressure cuff, positioned proximally around the upper arm to 50 mmHg. Blood flow was then measured by analyzing the increase of the slope of tHb, which is the sum of the O_2_Hb and HHb (in arbitrary units) [19].

#### Blood pressure

First, BP was measured twice at the left brachial artery using the standard auscultatory method with the volunteers in the supine position after a resting period of at least 10 min. Furthermore, arterial BP was measured continuously by a portable BP device (Nexfin; BMEYE B.V., Amsterdam, The Netherlands) connected to the middle phalanx of the index or middle finger of the left hand. Systemic vascular resistance (VR) and cardiac output (CO) were derived from the Nexfin finger volume-clamp method [20]. Continuous BP measurements were used to calculate local VR, which corrects muscle blood flow for BP fluctuations.

### Statistics

Because of the novelty of the study, the effect size of the interventions could not be estimated or calculated. Therefore we based the number of volunteers in the present explorative study on the sample size of earlier vascular studies [10, 21]. We adopted a Mixed Model Repeated measures ANCOVA with volunteers as the random factor to examine whether postprandial changes in blood flow and glucose homeostasis (i.e. fixed factor ‘time’) differed between the 3 interventions (fixed factor ‘intervention’). We included baseline values, age, BMI, waist-to-hip ratio, MAP and AUC insulin response (control) as covariates. Also the interaction time*intervention and baseline*intervention were included in the model. Each analysis started using the complete model. The model was then reduced by removal of non-significant factors, starting with non-significant interactions and then non-significant covariates. This process was guided by the AIC criterion (Akaike’s Information Criterion), the final model was the model with a minimum AIC. The covariates which contributed significantly to the model were baseline and BMI. The distribution of the residuals was found to have a positive skew for all measurement parameters. To comply with the Normality assumption of the ANCOVA method the natural logarithm of the parameter has been used in the analysis. Values reported are means ± SD per time point (tables), or back transformed Least-Squares Means (LSMeans) over all time points (text and tables), *p*-values were calculated for the differences in LSMeans compared to control. A *p*-value of less than 0.05 was considered significant. Descriptive statistics (table) are presented as median and Q1, Q3. Multiple testing was taken into account using the Tukey-Kramer method.

## Results

Sixteen male volunteers were included (Table 2). A data-check was performed before deblinding with predefined exclusion criteria: (i) data-points that were ≥2xSD off the individuals’ mean data; (ii) a data point if <1 min of data was recorded (technical error); (iii) measurements with missing data (technical error). Following completion of all measurements and data check, the randomisation order was revealed and the data was saved as the final data-set. We excluded glucose and insulin data for one measurement, echo-Doppler data for three measurements and NIRS data for six measurements out of 48 measurements.

**Table 2:** Overview of the participant characteristics at baseline

Variable	Median (Q1; Q3)
*n*	16
Age (years)	61 (56; 63)
Height (cm)	179 (175; 182)
Weight (kg)	106 (96; 111)
BMI (kg/m^2^)	32 (31; 34)
Waist circumference (cm)	112 (108; 115)
Waist-to-hip-ratio	1.03 (1.02; 1.05)
Cholesterol (mmol/l)	5.25 (4.49; 5.64)
Triacylglycerol (mmol/l)	1.96 (1.66; 2.55)
HDL (mmol/l)	1.19 (0.96; 1.42)
LDL (mmol/l)	3.4 (2.87; 4.08)
Screening glucose (mmol/l)	5.89 (5.76; 6.38)

*BMI*, body mass index

Values are median (Q1, Q3)

### Blood flow

**Fig. 1:**
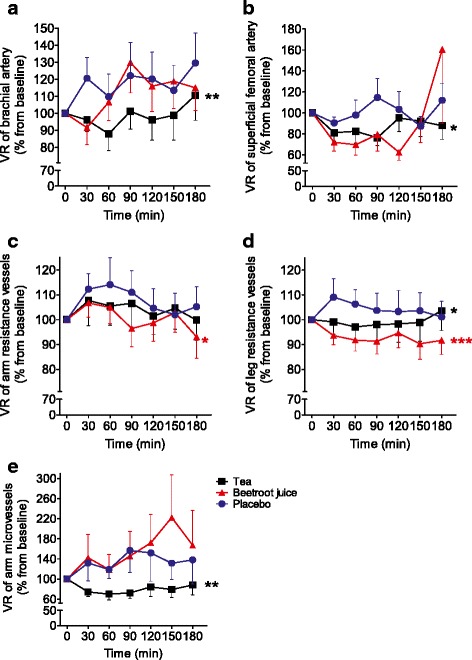
Effects of tea, beetroot juice and control on VR. VR was assessed by blood flow corrected for BP of conduit arteries at (**a**) arm and (**b**) leg, of resistance vessels at (**c**) arm and (**d**) leg and of microvessels (**e**) at the arm. Measurements were taken before and every 30-min (for 3-h) after 75-gr glucose intake in obese, insulin-resistant men (*n* = 16). Data are presented as mean ± SEM. * significant at *P* < 0.05; ** significant at *P* < 0.01; *** significant at *P* < 0.001 as compared to the mean over time of control treatment

Under control conditions, we observed in obese, insulin-resistant men no change in VR as assessed by blood flow corrected for BP after the glucose load independent of the vascular bed (Fig. 1a–e; Additional file 1: Table S1). Tea consumption reduced VR of conduit arteries (arm; *P* = 0.008 and leg; *P* = 0.033, Fig. 1a&b; Table 3), leg resistance vessels (*P* = 0.041, Fig. 1d; Table 3) and microvessels (arm, *P* = 0.002; Fig. 1e; Table 3), whilst arm resistance vessel did not change (*P* > 0.24, Fig. 1c; Table 3).

**Table 3:** Effects of interventions on VR of conduit arteries (echo-Doppler), resistance (VOP) and microvessels (NIRS)

Variable/treatment	Time (minutes)								
	−20	+30	+60	+90	+120	+150	+180	LSMeans	*P*-values
BA VR (echo-Doppler), mmHg ∙ min/ml									
Control	117 ± 59	127 ± 70	109 ± 49	119 ± 50	120 ± 52	116 ± 56	125 ± 52	100	
Beetroot juice	103 ± 57	89 ± 56	104 ± 66	137 ± 122	109 ± 57	123 ± 114	111 ± 61	98	ns
Black tea	98 ± 41	103 ± 58	88 ± 38	98 ± 44	92 ± 47	94 ± 56	106 ± 52	88	0.008
SFA VR (echo-Doppler), mmHg ∙ min/ml									
Control	61 ± 28	53 ± 31	58 ± 39	73 ± 69	61 ± 34	61 ± 55	74 ± 65	54	
Beetroot juice	87 ± 67	55 ± 22	49 ± 20	65 ± 56	48 ± 23	63 ± 49	90 ± 105	46	ns
Black tea	63 ± 69	60 ± 58	55 ± 55	51 ± 45	48 ± 32	107 ± 219	42 ± 17	46	0.033
Arm VR (VOP), mmHg ∙ min ∙ 100 ml tissue/ml									
Control	54 ± 22	60 ± 27	61 ± 36	62 ± 43	56 ± 28	54 ± 28	56 ± 31	50	
Beetroot juice	49 ± 23	53 ± 33	49 ± 20	44 ± 16	46 ± 20	47 ± 18	43 ± 23	44	0.020
Black tea	52 ± 17	55 ± 26	53 ± 22	53 ± 23	51 ± 18	52 ± 22	49 ± 18	48	ns
Leg VR (VOP), mmHg ∙ min ∙ 100ml tissue/ml									
Control	44 ± 27	45 ± 20	45 ± 27	45 ± 31	44 ± 31	44 ± 25	44 ± 31	40	
Beetroot juice	44 ± 23	42 ± 26	41 ± 27	41 ± 27	44 ± 33	41 ± 30	41 ± 30	35	<0.0001
Black tea	43 ± 19	43 ± 25	42 ± 25	43 ± 29	45 ± 36	43 ± 28	43 ± 22	38	0.041
Arm VR (NIRS), A.U.									
Control	129 ± 108	111 ± 66	132 ± 78	122 ± 49	155 ± 167	118 ± 92	124 ± 124	112	
Beetroot juice	152 ± 147	125 ± 89	121 ± 91	145 ± 125	170 ± 156	128 ± 101	118 ± 105	105	Ns
Black tea	201 ± 150	149 ± 142	125 ± 77	108 ± 53	133 ± 85	112 ± 69	117 ± 51	84	0.002

Values are means ± SD per time point or LSMeans over all time points, i.e. before (-20) and after 75 g glucose (at 30-min intervals for 3-h); *P*-values were calculated for the differences in LSMeans compared to control. *BA* brachial artery; ns: not significant, *SFA* superficial femoral artery, *VR* vascular resistance

The effect of beetroot juice was not consistent. The VR of resistance vessels was lowered after beetroot juice ingestion (arm, *P* = 0.0011 and leg, *P* < 0.0001; Fig. 1c&d; Table 3). However, beetroot juice did not reduce VR of conduit arteries (leg, *P* = 0.07; Fig. 1b, or arm, *P* = 0.60; Fig. 1a; Table 3) or microvessels (*P* = 0.41; Fig. 1e; Table 3).

#### Systemic vascular resistance and cardiac output

Neither SVR nor CO was significantly affected by tea (SVR: 744.6 mmHg l^−1^ min^−1^; CO: 11.1 l/min) or beetroot juice (SVR: 741.2 mmHg l^−1^ min^−1^; CO: 11.2 l/min) compared to control (SVR: 759.5 mmHg l^−1^ min^−1^; CO: 10.8 l/min). SVR was not significantly associated with either local leg or arm VR (data not shown).

#### Glucose homeostasis

**Fig. 2:**
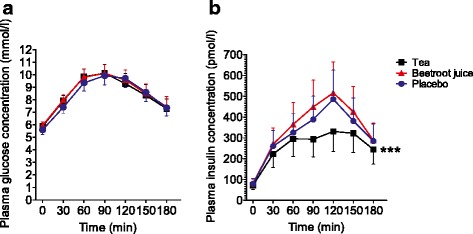
Effects of tea, beetroot juice and control on (**a**) plasma glucose and (**b**) insulin concentration. Measurements were taken before and every 30-min (for 3-h) after 75-gr glucose intake in obese, insulin-resistant men (*n* = 16). Data are presented as mean ± SEM. *** significant at *P* < 0.001 as compared to the mean over time of control treatment

Postprandial glucose showed a rapid increase, with plasma concentration doubled and reaching a maximum around 90 min after the glucose load. Although the glucose concentration gradually declined, it remained elevated for at least three hours (Fig. 2a). Tea or beetroot juice did not alter the postprandial changes in glucose plasma concentrations (*P* = 0.44 and *P* = 0.50; Fig. 2a).

Plasma insulin concentrations demonstrated an increase immediately after the glucose load with maximal values that peaked around 120 min, followed by a rapid decline (Fig. 2b). Tea ingestion resulted in a blunted postprandial increase in insulin, leading to a 29 % lower insulin area-under-the-curve of concentration versus time between 0 and 180 min compared to control (*P* < 0.0005; Fig. 2b). Beetroot juice had no significant impact on plasma insulin concentration after the glucose load (*P* = 0.48; Fig. 2b).

## Discussion

The present study provides a number of important observations. First, our study confirms previous observations that obese, insulin-resistant men demonstrate no change in VR response to a glucose load, which markedly contrasts the vasodilation typically observed in healthy individuals [4, 5]. Secondly, we found that tea resulted in a postprandial decrease in VR in conduit arteries, leg resistance vessels and microvessels, whereas beetroot juice led to a decrease in VR at the resistance vessels level only. Third, although postprandial glucose concentrations demonstrated a comparable, time-dependent change between all three interventions, tea ingestion was associated with a blunted postprandial increase in insulin. Taken together, these data suggest that single intake of tea results in an immediate improvement in postprandial blood flow and insulin sensitivity in response to a glucose load in obese, insulin-resistant men.

A potential explanation for the impact of tea on postprandial blood flow relates to the impact of tea on endothelial function. Several studies have established the benefit of tea on large vessel endothelial function [8]. Furthermore, consumption of tea protects against oral fat load-induced endothelial dysfunction in hypertensive volunteers [10]. These effects may relate to the presence of flavonoids in tea since ingestion of other flavonoid-rich food products (e.g. cocoa, red wine or berries) can improve endothelial function too. To support further evidence for a direct effect of tea on the vasculature, previous studies have provided evidence that consumption of green tea increased the blood flow in resistance vessels [22, 23] and microvessels [24]. Thus, tea enhances postprandial blood flow responses, possibly by improving endothelial function.

Tea also affected postprandial concentrations of insulin. This effect contrasts with findings in other studies that examined the impact of tea on postprandial glucose homeostasis, which may relate to differences in methodology (e.g. study population, tea-extract *vs* tea fraction, meal *vs* glucose) [25, 26]. The 29 % attenuation of the post-prandial insulin peak in the tea intervention may be related to the effects on postprandial blood flow. Results from clinical and experimental studies indicate that insulin stimulates the production of NO and thereby induces capillary recruitment and increases blood flow to the skeletal muscle, which contributes to glucose disposal [6]. Possibly, the postprandial increase in blood flow after tea ingestion required lower insulin concentrations for glucose uptake. At least in the fasted state, plasma insulin concentration and insulin resistance were significantly reduced without changes in fasting plasma glucose concentrations after 4 weeks of daily consumption of 100 mg (-)-epicatechin, with tea being the major dietary source of this flavonoid [27]. Despite changes in insulin, we found no impact of tea on post-prandial glucose levels. Plasma glucose concentration is determined by the balance of glucose entering the circulation (mediated by glucose absorption in the intestinal lumen, glycogenolysis, and gluconeogenesis) and glucose disposal [28]. Possibly, an immediate increase in glucose disposal via reduced VR and improved insulin sensitivity is counterbalanced by an increased release of glucose in the circulation through glycogenolysis and/or gluconeogenesis. Whether repeated exposure to such changes can alter glucose metabolism is currently unknown and should be subject for future research. At least, our observation suggests that flavonoid-rich tea ingestion may acutely affect insulin-sensitivity, possibly mediated via changes in postprandial blood flow.

Beetroot is particular rich in nitrate [29], which is an important source of NO [30], and therefore may affect the vasculature [31]. Since previous work found beetroot juice to attenuate the postprandial impairment of FMD [13], we expected beetroot juice to improve postprandial blood flow responses. Indeed, postprandial forearm and leg resistance artery blood flow was improved after beetroot juice ingestion. However, this was not accompanied by changes in conduit or microvessel blood flow, whilst we also found no effect on postprandial glucose or insulin concentrations. This inconsistency of the impact of beetroot juice on the different vascular beds may be caused by the difference in mechanisms involved in the vascular reactivity of macro- and microvasculature [32, 33]. The vasomotor responses to neural, metabolic and physical factors vary between vessels in different vascular beds and even along the same bed, particularly as vessels become smaller and the relative contribution of NO to vasodilation diminishes [33]. Of note, our findings are in line with previous studies in individuals with diabetes mellitus type 2 failing to show effects of beetroot juice supplementation on macrovascular or microvascular endothelial function, BP or insulin sensitivity [34–36]. The lack of effect may relate to the increased oxidative stress commonly seen in individuals with impaired glucose metabolism, resulting in a higher scavenging, and thus less bioactivity, of NO that is formed following nitrate supplementation [36].

In agreement with our findings, others did not detect an effect of inorganic nitrate on insulin or glucose after an oral glucose test or during an euglycemic clamp [37]. In contrast, Wootton-Beard et al. showed that beetroot juice lowered insulin and preserved glucose concentrations after a 50-gr carbohydrate meal [38]. A possible explanation for these findings may relate to the polyphenol, including flavonoids, content of the intervention products. The beetroot juice used in the study of Wootton-Beard et al. contained 326.3 mg polyphenols per serving [39], whilst beetroot juice and tea of the present study contained 68.4 and 228.1 mg polyphenols per serving, respectively. Possibly, larger amounts of polyphenols may relate to changes in postprandial blood flow and/or glucose homeostasis.

### Clinical relevance

Epidemiological data suggests that tea ingestion is associated with lower risk of diabetes mellitus type 2 [40]. A recent systematic review and dose-response meta-analysis of prospective cohort studies found a significant, inverse association between tea consumption and risk of developing diabetes mellitus type 2, with increased consumption of two cups of tea/day associated with a 4.6 % reduced risk of diabetes mellitus type 2 [41]. Our study provides the first potential insight in possible mechanisms by which tea may affect postprandial insulin sensitivity and, therefore, development and progression of diabetes mellitus type 2. Additional studies, preferably adopting randomized controlled clinical studies, on tea ingestion and glucose homeostasis are required to further explore the potential impact of tea ingestion.

#### Limitations

The current study has several strengths that give support to the conclusions, such as the within-volunteer design, inclusion of a tightly controlled population with impaired insulin resistance, and parallel measurements in different vascular beds in upper and lower limbs. However, due to the explorative character of the study, the study population was rather small. Both test products were too different in taste and color to design a single real placebo product and we used water as control. Therefore, the study was performed in a single-blinded way, with volunteers being aware of the test product identity.

## Conclusion

We observed that obese, insulin-resistant men demonstrate no change in postprandial blood flow, whilst postprandial plasma glucose and insulin concentrations increased significantly. Flavonoid-rich tea significantly decreased peripheral VR across upper and lower limb vascular beds, whereas insulin plasma concentrations were reduced without affecting plasma glucose. Beetroot juice showed less consistent effects on VR and no effect on glucose homeostasis. Taken together, flavonoid-rich tea may affect insulin sensitivity, possibly related to improvements in postprandial peripheral VR. Future studies should further explore the potential long-term clinical benefits of increasing the intake of flavonoid-rich tea on glucose homeostasis.

## Additional file

Additional file 1: Table S1 Effects of interventions on blood flow of conduit arteries (echo-Doppler), resistance (VOP) and microvessels (NIRS). (DOCX 19 kb)

## Abbreviations

BP: blood pressure
CO: cardiac output
FMD: flow-mediated dilation
HHb: desoxyhaemoglobin
NIRS: near-infrared spectroscopy
NO: nitric oxide
O_2_Hb: oxyhaemoglobin
SVR: systemic vascular resistance
tHb: total hemoglobin signal
VOP: venous occlusion plethysmography
VR: vascular resistance

## References

[1] American Diabetes Association (2001). Postprandial Blood Glucose. Clin Diab.

[2] Thiebaud D, Jacot E, Defronzo RA, Maeder E, Jequier E, Felber JP (1982). The effect of graded doses of insulin on total glucose uptake, glucose oxidation, and glucose storage in man. Diabetes.

[3] Vincent MA, Clerk LH, Lindner JR, Klibanov AL, Clark MG, Rattigan S (2004). Microvascular recruitment is an early insulin effect that regulates skeletal muscle glucose uptake in vivo. Diabetes.

[4] Clark MGW (2003). Blood flow and muscle metabolism: a focus on insulin action. Am J Physiol Endocrinol Metab.

[5] Mitrou P, Boutati E, Lambadiari V, Maratou E, Papakonstantinou A, Komesidou V (2009). Rates of glucose uptake in adipose tissue and muscle in vivo after a mixed meal in women with morbid obesity. J Clin Endocrinol Metab.

[6] Kim JA, Montagnani M, Koh KK, Quon MJ (2006). Reciprocal relationships between insulin resistance and endothelial dysfunction: molecular and pathophysiological mechanisms. Circulation.

[7] Stensvold I, Tverdal A, Solvoll K, Foss OP (1992). Tea consumption. Relationship to cholesterol, blood pressure, and coronary and total mortality. Prev Med.

[8] Ras RT, Zock PL, Draijer R (2011). Tea consumption enhances endothelial-dependent vasodilation; a meta-analysis. PLoS One.

[9] Grassi D, Desideri G, Di Giosia P, De Feo M, Fellini E, Cheli P (2013). Tea, flavonoids, and cardiovascular health: endothelial protection. Am J Clin Nutr.

[10] Grassi D, Draijer R, Desideri G, Mulder T, Ferri C (2015). Black tea lowers blood pressure and wave reflections in fasted and postprandial conditions in hypertensive patients: a randomised study. Nutrients.

[11] Lundberg JO, Feelisch M, Bjorne H, Jansson EA, Weitzberg E (2006). Cardioprotective effects of vegetables: Is nitrate the answer?. Nitric Oxide.

[12] Bahra M, Kapil V, Pearl V, Ghosh S, Ahluwalia A (2012). Inorganic nitrate ingestion improves vascular compliance but does not alter flow-mediated dilatation in healthy volunteers. Nitric Oxide.

[13] Joris PJ, Mensink RP (2013). Beetroot juice improves in overweight and slightly obese men postprandial endothelial function after consumption of a mixed meal. Atherosclerosis.

[14] Alberti KG, Eckel RH, Grundy SM, Zimmet PZ, Cleeman JI, Donato KA (2009). Harmonizing the metabolic syndrome: a joint interim statement of the international diabetes federation task force on epidemiology and prevention; national heart, lung, and blood institute; American heart association; world heart federation; international atherosclerosis society; and international association for the study of obesity. Circulation.

[15] Assmann G, Guerra R, Fox G, Cullen P, Schulte H, Willett D (2007). Harmonizing the definition of the metabolic syndrome: comparison of the criteria of the adult treatment panel iii and the international diabetes federation in United States American and European populations. Am J Cardiol.

[16] Thijssen DH, Black MA, Pyke KE, Padilla J, Atkinson G, Harris RA (2011). Assessment of flow-mediated dilation in humans: a methodological and physiological guideline. Am J Physiol Heart Circ Physiol.

[17] Joyner MJ, Dietz NM, Shepherd JT (2001). From Belfast to Mayo and beyond: the use and future of plethysmography to study blood flow in human limbs. J Appl Physiol (1985).

[18] Livera LN, Wickramasinghe YA, Spencer SA, Rolfe P, Thorniley MS (1992). Cyclical fluctuations in cerebral blood volume. Arch Dis Child.

[19] Brunnekreef JJ, Oosterhof J, Thijssen DH, Colier WN, van Uden CJ (2006). Forearm blood flow and oxygen consumption in patients with bilateral repetitive strain injury measured by near-infrared spectroscopy. Clin Physiol Funct Imaging.

[20] Martina JR, Westerhof BE, Van GJ, de Beaumont EM, Truijen J, Kim YS (2012). Noninvasive continuous arterial blood pressure monitoring with Nexfin(R). Anesthesiology.

[21] Thijssen DH, Bleeker MW, Smits P, Hopman MT (2005). Reproducibility of blood flow and post-occlusive reactive hyperaemia as measured by venous occlusion plethysmography. Clin Sci (Lond).

[22] Oyama J, Maeda T, Kouzuma K, Ochiai R, Tokimitsu I, Higuchi Y (2010). Green tea catechins improve human forearm endothelial dysfunction and have antiatherosclerotic effects in smokers. Circ J.

[23] Nagaya N, Yamamoto H, Uematsu M, Itoh T, Nakagawa K, Miyazawa T (2004). Green tea reverses endothelial dysfunction in healthy smokers. Heart.

[24] Heinrich U, Moore CE, De Spirt S, Tronnier H, Stahl W (2011). Green tea polyphenols provide photoprotection, increase microcirculation, and modulate skin properties of women. J Nutr.

[25] Bryans JA, Judd PA, Ellis PR (2007). The effect of consuming instant black tea on postprandial plasma glucose and insulin concentrations in healthy humans. J Am Coll Nutr.

[26] Louie JC, Atkinson F, Petocz P, Brand-Miller JC (2008). Delayed effects of coffee, tea and sucrose on postprandial glycemia in lean, young, healthy adults. Asia Pac J Clin Nutr.

[27] Dower JI, Geleijnse JM, Gijsbers L, Zock PL, Kromhout D, Hollman PC (2015). Effects of the pure flavonoids epicatechin and quercetin on vascular function and cardiometabolic health: a randomized, double-blind, placebo-controlled, crossover trial. Am J Clin Nutr.

[28] Aronoff SL, Berkowitz K, Shreiner B, Want L (2004). Glucose metabolism and regulation: beyond insulin and glucagon. Diab Spectr.

[29] Siervo M, Lara J, Ogbonmwan I, Mathers JC (2013). Inorganic nitrate and beetroot juice supplementation reduces blood pressure in adults: a systematic review and meta-analysis. J Nutr.

[30] Bos PM, Van den Brandt PA, Wedel M, Ockhuizen T (1988). The reproducibility of the conversion of nitrate to nitrite in human saliva after a nitrate load. Food Chem Toxicol.

[31] Kapil V, Khambata RS, Robertson A, Caulfield MJ, Ahluwalia A (2015). Dietary nitrate provides sustained blood pressure lowering in hypertensive patients: a randomized, phase 2, double-blind, placebo-controlled study. Hypertension.

[32] Minson CT, Green DJ (2008). Measures of vascular reactivity: prognostic crystal ball or Pandora’s box?. J Appl Physiol (1985).

[33] Hill CE, Phillips JK, Sandow SL (2001). Heterogeneous control of blood flow amongst different vascular beds. Med Res Rev.

[34] Cermak NM, Hansen D, Kouw IWK, van Dijk JW, Blackwell JR, Jones AM (2015). A single dose of sodium nitrate does not improve oral glucose tolerance in patients with type 2 diabetes mellitus. Nutr Res.

[35] Gilchrist M, Winyard PG, Aizawa K, Anning C, Shore A, Benjamin N (2013). Effect of dietary nitrate on blood pressure, endothelial function, and insulin sensitivity in type 2 diabetes. Free Radic Biol Med.

[36] Shepherd AI, Gilchrist M, Winyard PG, Jones AM, Hallmann E, Kazimierczak R (2015). Effects of dietary nitrate supplementation on the oxygen cost of exercise and walking performance in individuals with type 2 diabetes: a randomized, double-blind, placebo-controlled crossover trial. Free Radic Biol Med.

[37] Larsen FJ, Schiffer TA, Ekblom B, Mattsson MP, Checa A, Wheelock CE (2014). Dietary nitrate reduces resting metabolic rate: a randomized, crossover study in humans. Am J Clin Nutr.

[38] Wootton-Beard PC, Brandt K, Fell D, Warner S, Ryan L (2014). Effects of a beetroot juice with high neobetanin content on the early-phase insulin response in healthy volunteers. J Nutr Sci.

[39] Wootton-Beard PC, Ryan L (2011). A beetroot juice shot is a significant and convenient source of bioaccessible antioxidants. J Funct Foods.

[40] Huxley R, Lee CM, Barzi F, Timmermeister L, Czernichow S, Perkovic V (2009). Coffee, decaffeinated coffee, and tea consumption in relation to incident type 2 diabetes mellitus: a systematic review with meta-analysis. Arch Intern Med.

[41] Yang WS, Wang WY, Fan WY, Deng Q, Wang X (2014). Tea consumption and risk of type 2 diabetes: a dose-response meta-analysis of cohort studies. Br J Nutr.

