# Supplementary material for: Impact of flavonoid-rich black tea and beetroot juice on postprandial peripheral vascular resistance and glucose homeostasis in obese, insulin-resistant men: a randomized controlled trial
Source: Nutr Metab (Lond). 2016 May 13;13:34. doi: 10.1186/s12986-016-0094-x (PMC4866334; doi:10.1186/s12986-016-0094-x)
Supplement: Additional file 1: Table S1 — Effects of interventions on blood flow of conduit arteries (echo-Doppler), resistance (VOP) and microvessels (NIRS). (DOCX 19 kb) [file 12986_2016_94_MOESM1_ESM.docx]

**Additional file 1: Table S1.** Effects of interventions on blood flow of conduit arteries (echo-Doppler), resistance (VOP) and microvessels (NIRS).

| **Variable/treatment** | **Time (minutes)** | | | | | |  | | |
| --- | --- | --- | --- | --- | --- | --- | --- | --- | --- |
|  | -20 | +30 | +60 | +90 | +120 | +150 | +180 | LSMeans | P-values |
| BA blood flow (echo-Doppler), *ml/min* | |  |  |  |  |  |  |  |  |
| Control | 1.1 ± 0.6 | 1.1 ± 0.7 | 1.2 ± 0.8 | 1.1 ± 0.7 | 1.1 ± 0.6 | 1.1 ± 0.6 | 1.0 ± 0.5 | 1.0 |  |
| Beetroot juice | 1.3 ± 0.6 | 1.5 ± 0.8 | 1.3 ± 0.5 | 1.2 ± 0.6 | 1.2 ± 0.6 | 1.3 ± 0.6 | 1.2 ± 0.5 | 1.1 | ns |
| Black tea | 1.2 ± 0.6 | 1.3 ± 0.8 | 1.4 ± 0.6 | 1.3 ± 0.7 | 1.4 ± 0.7 | 1.5 ± 0.8 | 1.3 ± 0.7 | 1.2 | 0.003 |
| SFA blood flow (echo-Doppler), *ml/min* | |  |  |  |  |  |  |  |  |
| Control | 1.9 ± 0.8 | 2.3 ± 0.9 | 2.3 ± 1.1 | 2.1 ± 1.0 | 2.2 ± 1.3 | 2.7 ± 1.5 | 2.3 ± 1.2 | 1.8 |  |
| Beetroot juice | 1.6 ± 0.7 | 2.1 ± 0.8 | 2.5 ± 1.1 | 2.5 ± 1.5 | 2.7 ± 1.4 | 2.4 ± 1.2 | 2.4 ± 1.7 | 2.2 | 0.039 |
| Black tea | 2.2 ± 0.8 | 2.6 ± 1.3 | 2.7 ± 1.3 | 3.1 ± 1.6 | 3.1 ± 2.0 | 3.0 ± 1.8 | 3.1 ± 1.7 | 2.2 | 0.015 |
| Arm BF (VOP), *ml 100 ml^-1^ min^-1^* | |  |  |  |  |  |  |  |  |
| Control | 2.2 ± 0.8 | 2.0 ± 0.9 | 2.1 ± 1.0 | 2.2 + 1.1 | 2.3 ± 1.1 | 2.3 ± 1.1 | 2.4 ± 1.1 | 2.0 |  |
| Beetroot juice | 2.5 ± 1.3 | 2.4 ± 1.2 | 2.5 ± 1.1 | 2.7 ± 1.2 | 2.7 ± 1.2 | 2.6 ± 1.0 | 2.9 ± 1.0 | 2.3 | 0.028 |
| Black tea | 2.2 ± 1.1 | 2.3 ± 1.2 | 2.3 ± 1.1 | 2.3 ± 1.2 | 2.3 ± 1.1 | 2.3 ± 0.9 | 2.4 ± 0.9 | 2.1 | ns |
| Leg BF (VOP), *ml 100 ml^-1^ min^-1^* | |  |  |  |  |  |  |  |  |
| Control | 2.8 ± 1.2 | 2.6 ± 0.9 | 2.7 ± 1.1 | 2.8 ± 1.1 | 2.9 ± 1.2 | 2.8 ± 1.2 | 3.1 ± 1.4 | 2.6 |  |
| Beetroot juice | 2.9 ± 1.7 | 3.2 ± 2.2 | 3.4 ± 2.3 | 3.4 ± 2.1 | 3.5 ± 2.5 | 3.7 ± 2.5 | 3.4 ± 1.9 | 2.9 | <0.0001 |
| Black tea | 2.9 ± 1.6 | 3.1 ± 2.1 | 3.2 ± 2.1 | 3.2 ± 2.0 | 3.3 ± 2.2 | 3.3 ± 2.0 | 3.1 ± 1.7 | 2.7 | 0.016 |
| Arm BF (NIRS), *A.U.* |  |  |  |  |  |  |  |  |  |
| Control | 1.4 ± 1.2 | 1.2 ± 0.6 | 1.0 ± 0.4 | 1.1 ± 1.0 | 1.4 ± 1.2 | 1.4 ± 1.2 | 1.4 ± 1.0 | 0.9 |  |
| Beetroot juice | 1.6 ± 1.5 | 1.5 ± 1.5 | 1.5 ± 1.4 | 1.3 ± 1.0 | 1.1 ± 0.7 | 1.4 ± 1.2 | 1.6 ± 1.1 | 1.0 | ns |
| Black tea | 0.9 ± 0.7 | 1.3 ± 1.1 | 1.3 ± 1.1 | 1.2 ± 0.8 | 1.3 ± 1.1 | 1.4 ± 0.9 | 1.1 ± 0.5 | 1.2 | 0.001 |

Values are means±SD per time point or LSMeans over all time points, i.e. before (-20) and after 75 g glucose (at 30-minute intervals for 3-h); P-values were calculated for the differences in LSMeans compared to control. BA: brachial artery; BF: blood flow; ns: not significant; SFA: superficial femoral artery
